# Editorial: Global excellence in nutritional epidemiology

**DOI:** 10.3389/fnut.2023.1195670

**Published:** 2023-07-20

**Authors:** Donato Angelino, Daniela Martini, Monica Dinu

**Affiliations:** ^1^Department of Bioscience and Technology for Food, Agriculture and Environment, University of Teramo, Teramo, Italy; ^2^Department of Food, Environmental and Nutritional Sciences (DeFENS), University of Milan, Milan, Italy; ^3^Department of Experimental and Clinical Medicine, University of Florence, Florence, Italy

**Keywords:** nutritional epidemiology, diet, vitamin, dietary patterns, food intake

Global collaboration is the cornerstone of scientific advancement. For this reason, Frontiers in Nutrition has organized a series of special edition Research Topics, with the goal of highlighting the latest advancements in Nutritional Epidemiology across the globe, showcasing the academic excellence and high-quality work of internationally recognized researchers. These collections aimed to shed light on the recent progress made across the entire breadth of the Nutritional Epidemiology field and reflect on the future challenges faced by researchers across borders.

A particular topic that was considered in depth in the present Research Topic was vitamin D deficiency in wide cohorts of individuals. Specifically, Cui, Zhang et al. retrieved data from 308 studies conducted in 81 countries of the world, involving almost 8 million individuals. Data highlighted a high prevalence of vitamin D deficiency, mainly in the Eastern Mediterranean region and lower-middle-income countries during the winter-spring time, and it affected mainly women. These data have been confirmed by research on US adults belonging to *The National Health and Nutrition Examination Surveys* (NHANES) cohort in a study led by the same authors (Cui, Xiao et al.). Specifically, the authors concluded that some aspects, both non-modifiable (i.e., age, gender, ethnicity, and season) and modifiable (i.e., sun-protective behaviors, lower BMI, lower socioeconomic status, drinking, and lower milk consumption) were predictors of severe vitamin D deficiency.

Another interesting study focusing on adolescents of the UK *National Diet and Nutrition Survey* program was led by Bawajeeh et al. who considered the importance of the taste of food in nutritional choices and, in turn, in the dietary intake of 289 UK individuals aged 10 to 19 years. Results showed that adolescents had a high intake of sweet-tasting foods, particularly during breakfast and snack time. In addition, authors showed that the increased consumption of sweet foods also led to increased intake of not only sugar but also fat and salt.

Finally, Dinu et al. retrieved data from diet-related clinical trials registered on the *National Institute of Health “ClinicalTrials.gov”* web platform over the past 10 years to provide an overview of the main considered dietary patterns in nutritional intervention research. The authors analyzed 1016 clinical trials, finding that the most studied diets in the world were balanced diets, those based on macronutrient modification (e.g., ketogenic diet), and those based on time-restricted feeding. The authors also found that most studies were conducted only among overweight/obese volunteers, and very few studies considered older adults.

In conclusion, all these studies emphasized the importance of nutritional epidemiology studies in identifying pitfalls in dietary patterns, which in turn affect the health of individuals. In particular, some nutrient deficiencies and the nutrition education of the young generation have been pointed out as a priority topic to be considered by public entities, stakeholders, and clinical professionals in order to counteract the insurgence and the diffusion of chronic metabolic diseases.

## Author contributions

All authors listed have made a substantial, direct, and intellectual contribution to the work and approved it for publication.

